# Suppression of Liquid‐Liquid Phase Separation and Aggregation of Antibodies by Modest Pressure Application

**DOI:** 10.1002/chem.202201658

**Published:** 2022-07-18

**Authors:** Zamira Fetahaj, Michel W. Jaworek, Rosario Oliva, Roland Winter

**Affiliations:** ^1^ Physical Chemistry I–Biophysical Chemistry Department of Chemistry and Chemical Biology TU Dortmund Otto-Hahn-Strasse 4a 44227 Dortmund Germany; ^2^ Department of Chemical Sciences University of Naples Federico II Via Cintia 4 80126 Naples Italy

**Keywords:** antibodies, excipients, high pressure, liquid-liquid phase separation, protein condensates

## Abstract

The high colloidal stability of antibody (immunoglobulin) solutions is important for pharmaceutical applications. Inert cosolutes, excipients, are generally used in therapeutic protein formulations to minimize physical instabilities, such as liquid–liquid phase separation (LLPS), aggregation and precipitation, which are often encountered during manufacturing and storage. Despite their widespread use, a detailed understanding of how excipients modulate the specific protein‐protein interactions responsible for these instabilities is still lacking. In this work, we demonstrate the high sensitivity to pressure of globulin condensates as a suitable means to suppress LLPS and subsequent aggregation of concentrated antibody solutions. The addition of excipients has only a minor effect. The high pressure sensitivity observed is due to the fact that these flexible Y‐shaped molecules create a considerable amount of void volume in the condensed phase, leading to an overall decrease in the volume of the system upon dissociation of the droplet phase by pressure already at a few tens of to hundred bar. Moreover, we show that immunoglobulin molecules themselves are highly resistant to unfolding under pressure, and can even sustain pressures up to about 6 kbar without conformational changes. This implies that immunoglobulins are resistant to the pressure treatment of foods, such as milk, in high‐pressure food‐processing technologies, thereby preserving their immunological activity.

## Graphical abstract

**Hydrostatic pressures** of a few tens of to hundred bar are sufficient to suppress liquid–liquid phase separation and subsequent aggregation and fibril formation of immunoglobulins. The immunoglobulin molecules themselves are highly resistant to unfolding under pressure, however, so the immunological activity of foods processed under high pressure is preserved.

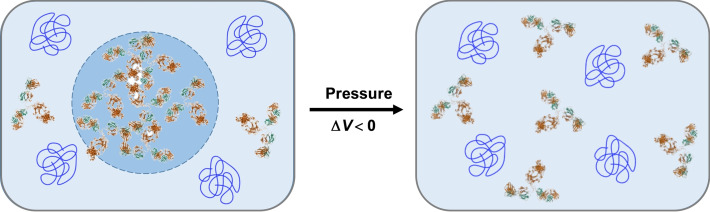

## Introduction

Antibodies, also known as immunoglobulins (Igs), in particular IgGs, are among the most important therapeutics due to their high specificity and low toxicity. They have revolutionized the treatment of more than a few human diseases, including cancer, autoimmunity, inflammatory and infectious conditions.[^1^, ^2^] In the last two years, a large number of monoclonal antibodies have also been developed to fight COVID‐19.^[3]^ Unfortunately, antibodies (Abs) are only marginally thermodynamically stable and often need to be formulated at high concentrations, rendering them susceptible to phase separation, aggregation, and precipitation. The concentration of total IgG in blood is normally within 10–25 mg mL^−1^. Concentrated IgG solutions are often needed in pharmaceutical applications to achieve the desired therapeutic effect. In such cases, antibody drugs are stored and administered in concentrations up to about 100 mg mL^−1^.[^1^, ^2^] To maintain the stability and shelf‐life of Abs and thus save these expensive protein‐based therapeutics, two strategies are often employed. On the one hand, the protein sequence may be altered, on the other hand, extrinsic factors such as the solvent conditions may be changed by adding cosolutes (excipients) which affect protein‐protein interactions and hence the stability of the protein formulation.[^4^, ^5^, ^6^]

At high concentrations, immunoglobulins undergo liquid‐liquid phase separation (LLPS) at low temperatures, that is, they phase separate into protein‐poor and protein‐rich liquid phases, in particular when formulated at low ionic strength and buffered at neutral pH near their isoelectric point.[^2^, ^4^, ^5^, ^6^, ^7^, ^8^, ^9^, ^10^] It is often observed that such fluid‐like droplet phases undergo liquid‐to‐solid gel‐like phase transitions over time, which upon maturation (or expedited by disease‐associated mutations) lead to fibril formation and the development of pathological diseases, such as Parkinson's, Alzheimer's, cataract, and antibody light‐chain (AL) amyloidosis.[^11^, ^12^] In AL amyloidosis, fibrils are deposited in various organs, most often in the heart and kidney, and impair their function.^[12]^ LLPS is generally driven by weak multivalent interactions, such as electrostatic, hydrophobic, π‐π and cation–π interactions,[^13^, ^14^] and strongly affected by external conditions including temperature, pH, ionic strength, and the types and concentrations of excipients. Recently, we and others observed that protein systems undergoing LLPS can be very sensitive to pressure,[^15^, ^16^, ^17^, ^18^, ^19^, ^20^, ^21^, ^22^] thus suggesting that pressure modulation may be used to suppress LLPS formation and subsequent irreversible aggregation and fibrilization. Generally, pressure is a mild perturbing agent that acts instantaneously and uniformly and is very sensitive to volumetric properties, so no additional mixing is needed. Pressure ramps can be applied in both phase transition directions without changes in sample composition and pressure‐induced changes are generally fully reversible.[^23^, ^24^, ^25^, ^26^]

In this work, we explored the effect of pressure on γ‐globulin, a polyvalent antibody mixture consisting of IgG, IgM and IgA, as a model immunoglobulin system that undergoes liquid–liquid phase separation, and whose temperature‐concentration dependent phase behavior has been characterized, recently.[^5^, ^7^, ^9^] The main component of γ‐globulin is IgG (∼80 %), a rather flexible, nonspherical Y‐shaped protein which consists of four disulfide‐linked peptide chains, two heavy chains of about 55 kDa and two light chains of about 20 kDa. To determine the pressure dependent phase behavior and structure of the system, light microscopy, FTIR, UV/Vis absorption and fluorescence spectroscopies were applied using high‐pressure sample cells, complemented by calorimetric studies. In the presence of the nonionic crowding agent poly(ethylene glycol) (PEG), a typical agent mimicking intracellular crowding effects, the attraction between the protein molecules increases isotropically through the Asakura–Oosawa depletion interaction, giving rise to phase separation even at room temperature. Depletion forces originate from steric exclusion of PEG from the contact area between the protein molecules and are of entropic nature.[^27^, ^28^] As organic cosolvents are common excipients used to control the colloidal stability of concentrated antibody solutions, we studied also the impact of trimethylamine‐*N*‐oxide (TMAO) on the stability of the droplet phase of γ‐globulin. TMAO is a very effective compatible osmolyte which is upregulated in organisms thriving in the deep sea at high pressures of several hundred bar to help stabilize proteins and their functions under such harsh environmental conditions.[^29^, ^30^, ^31^, ^32^, ^33^, ^34^]

## Results and Discussion

**Figure 1:**
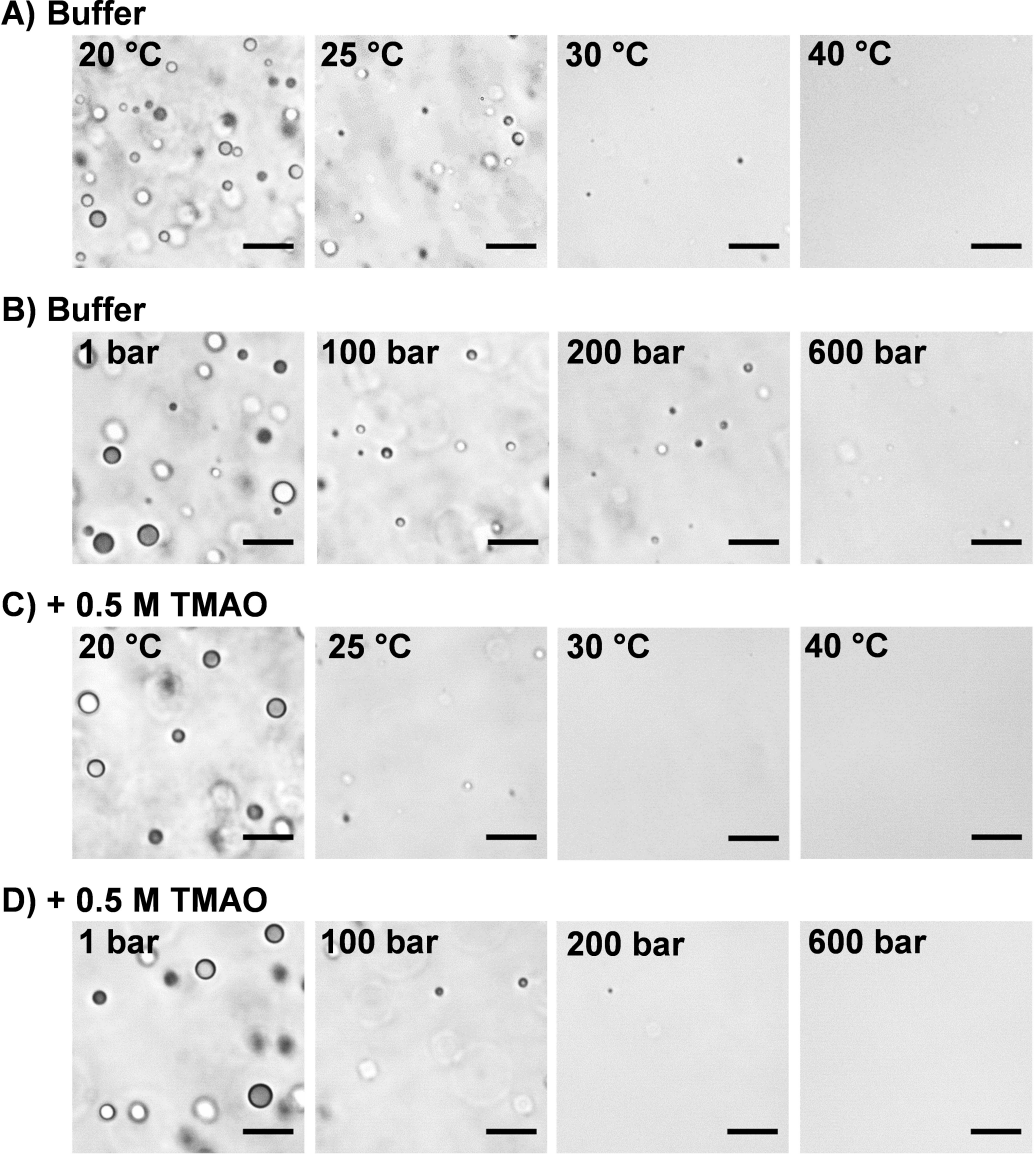
Light microscopy snapshots of 80 mg mL^−1^ γ‐globulin in 10 % (*w*/*v*) PEG 1000 containing buffer solution as a function of A) temperature and B) pressure at *T*=20 °C, and in the presence of 0.5 M TMAO as a function of C) temperature and D) pressure dependence at *T*=20 °C. Scale bars: 20 μm.

To visualize the temperature, pressure and cosolvent dependent phase behavior of the γ‐globulin system, bright‐field light microscopy studies were carried out. Figure 1 shows light microscopy snapshots of a 80 mg mL^−1^ γ‐globulin/10 % (*w/v*) PEG 1000 solution in neat buffer and in 0.5 M TMAO at selected temperatures and 1 bar as well as at selected pressures for *T*=20 °C. Under the light microscope, micrometer‐sized droplets were clearly visible at low temperatures, which are characteristic for the liquid‐liquid phase separated state, and they completely dissolved when the solution temperature was increased above about 30 °C. We also investigated the effect of pressure and temperature on γ‐globulin droplets sitting directly on the bottom window of the microscopy cell under both solution conditions. The results (Figure S1 in the Supporting Information) indicate that interfacial wetting has a little stabilizing effect on the droplet phase, only. The general sensitivity of the LLPS system to elevated temperatures and pressures remained.

**Figure 2:**
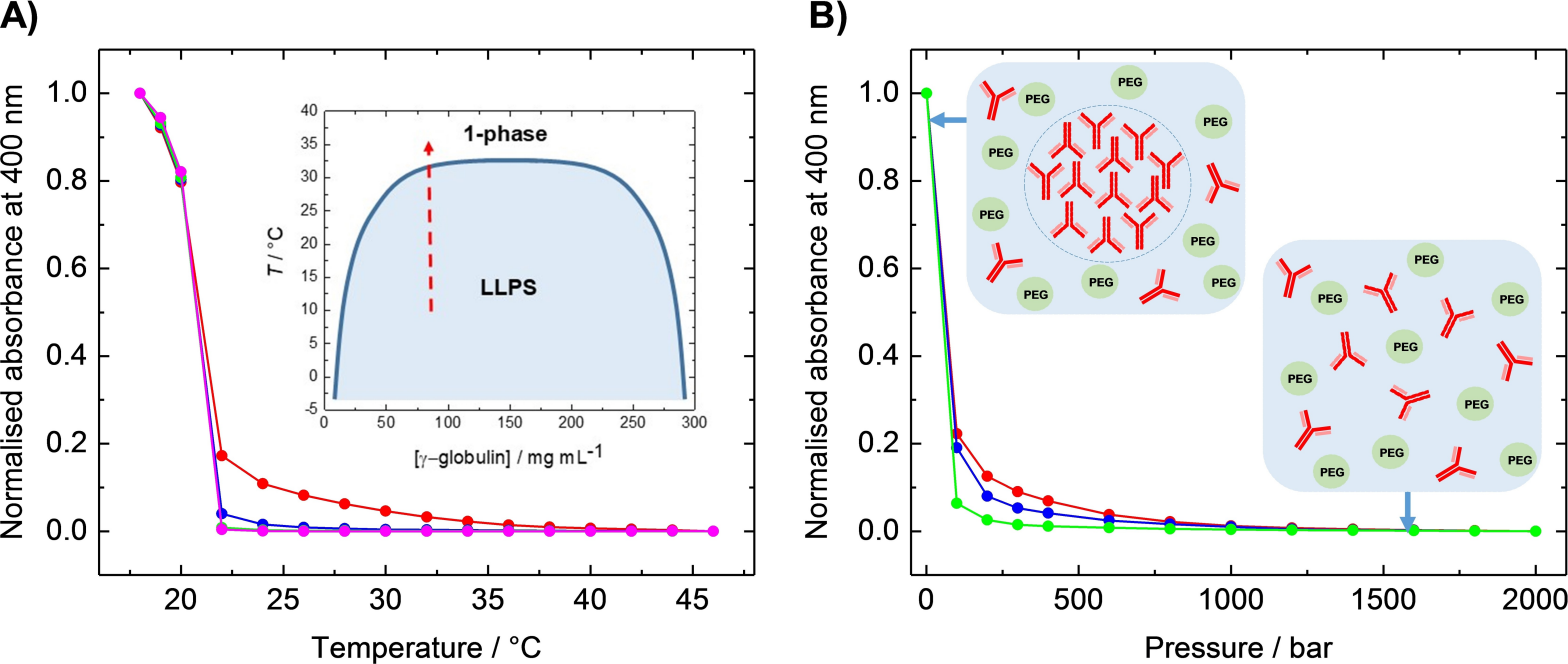
A) UV‐Vis absorption at 400 nm of 80 mg mL^−1^ γ‐globulin/10 % (*w*/*v*) PEG 1000 as a function of temperature in buffer (red), 0.2 M TMAO (blue), 0.5 M TMAO (green), and 0.7 M TMAO (magenta). The absorption data were normalized to 1.0 representing their maximum values. Data points are averages of three independent measurements. Below a particular temperature, the “cloud temperature” (*T*_cloud_), the sample became visibly cloudy, and the transmitted intensity rapidly dropped, leading to a sharp increase in the absorption. This clouding marks the onset of phase separation and is due to the formation of small droplets of protein‐rich phase in the dilute solution or small droplets of protein‐poor phase in the concentrated protein solution. Inset: Temperature‐concentration phase diagram of γ‐globulin/10 % (*w*/*v*) PEG 1000^[7]^ and direction of the turbidity measurement (red arrow). B) UV‐Vis absorption at 400 nm of γ‐globulin/10 % (*w*/*v*) PEG 1000 as a function of pressure in buffer (red), 0.2 M TMAO (blue), and 0.5 M TMAO (green) at *T*=20 °C. Inset: Schematic pictures representing the PEG‐induced liquid–liquid phase separation (LLPS) region of γ‐globulin (red) and the dissolution of the liquid droplet phase at high pressures.

Complementary absorption (turbidity) measurements were carried out at 400 nm using a UV/Vis spectrometer to more accurately localize phase transition temperatures (i. e., the cloud‐point temperatures, *T*_cloud_) and pressures (i. e., the cloud point pressures, *p*_cloud_) when entering and exiting the two‐phase region. The pressure‐dependent absorption measurements were carried out using a home‐built high‐pressure optical cell.[^15^, ^16^, ^17^] Bright‐field light microscopy using a high‐pressure diamond cell was applied to visualize the formation and dissolution of protein droplets on the μm scale (see the Methods section in the Supporting Information for experimental details).[^15^, ^16^, ^17^] At *T*_cloud_ or *p*_cloud_, the samples became cloudy and the transmitted light intensity rapidly changed, indicating crossing the conodal (coexistence) curve. In the presence of 10 % (*w*/*v*) PEG 1000, the protein phase separates below a temperature of ∼30 °C, in agreement with literature data (see inset of Figure 2A)^[7]^ and the microscopy results. Adding TMAO destabilizes the protein's LLPS slightly, shifting the onset of phase separation down to ∼22 °C. This effect was already observed at TMAO concentrations as low as 0.2 M. Application of pressure at 20 °C dissolved the protein droplets completely at about 400 bar in buffer solution, indicating disappearance of the LLPS region at rather low pressures. Already a few 10 to 100 bar are sufficient to drastically reduce the amount of the droplet phase (Figure 2B), however. Overall, the results of the turbidity measurements are in good agreement with those of the light microscopy measurements (Figure 1) in terms of the location of the LLPS stability region. Again, the addition of TMAO was seen to destabilize the phase‐separated protein solution, lowering the cloud point pressure in a concentration‐dependent manner. A different scenario was observed in other proteinaceous LLPS systems. For example, TMAO has a stabilizing effect on the droplet phase of SynGAP/PSD‐95, a model LLPS system for postsynaptic densities, likely due in large part to its exclusion from the protein interface, which favors compact structures, including protein‐rich droplets.^[20]^

To gain insight into how temperature and TMAO affect the folded state of γ‐globulin, we performed differential scanning calorimetry (DSC) measurements as any changes in protein stability would also affect the location of the coexistence curve of the LLPS region. DSC measurements in pure buffer revealed an unfolding/denaturation temperature of the protein of *T*_m_=73.9±0.1 °C. The addition of 0.5 M TMAO had a minor stabilizing effect on the protein (in terms of *T*_m_ values), yielding a *T*_m_ value of 75.4±0.1 °C (Figure S2). Such behavior might originate in TMAO′s effect of increasing the hydrogen‐bonding network structure of water, resulting in preferential hydration and general stabilization of proteins in TMAO/water solutions.[^30^, ^31^, ^32^, ^33^] Conversely, addition of TMAO lead to a slight destabilization of the droplet phase of the system. A similar observation was made by Banks and Cordia, who also found a destabilizing effect of multiple cosolutes on the temperature‐dependent LLPS of monoclonal antibodies which did not correlate with the structural temperature stability as determined by DSC.^[10]^

To yield a better understanding of the mechanism by which pressure and the cosolvent affect the dissolution of the droplet phase of γ‐globulin, steady‐state fluorescence anisotropy measurements were employed to determine the binding constant, *K*_b_, of γ‐globulin molecules upon complex formation. The pressure‐dependent binding assay was performed by measuring the fluorescence anisotropy of dansyl‐labeled γ‐globulin in pure buffer and in the presence of 0.5 M TMAO. The binding isotherms were obtained by plotting the fluorescence anisotropy change, Δ*r=r*−*r*_0_ (where *r* and *r*_0_ are the anisotropies of labeled γ‐globulin in the presence and in the absence of unlabeled γ‐globulin, respectively), versus the total concentration of γ‐globulin, [γ‐globulin]_total_. The experimental data were fitted assuming that one γ‐globulin can interact with another γ‐globulin forming a dimer. This simple binding model was used because the stoichiometry of complex formation is unknown and the data could be well fitted with this model. The conclusions drawn from the binding studies, that is, the small pressure effect on the binding constant, which most likely cannot be responsible for the high pressure sensitivity of the system (see below), is not affected if the binding geometry were different.

**Figure 3:**
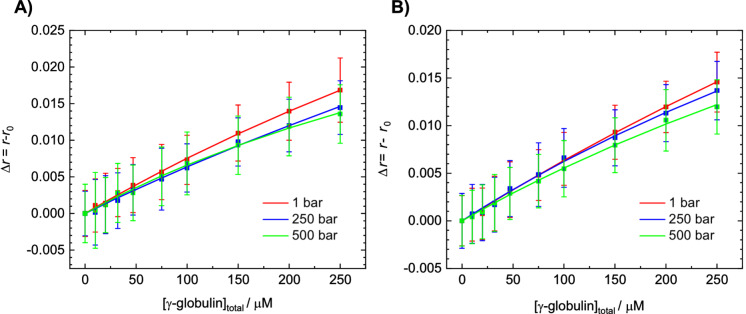
Fluorescence anisotropy data for the self‐association of γ‐globulin at different pressures A) in buffer and B) in the presence of 0.5 M TMAO at *T*=20 °C. The experimental data, Δ*r* vs. [γ‐globulin]_total_, were fitted accordingly to a 1 : 1 binding model (represented by lines) in order to evaluate the binding constant, *K*_b_.

Figure 3 shows the binding isotherms obtained at ambient temperature (20 °C) and selected pressures in neat buffer and in 0.5 M TMAO, respectively. As expected, the binding constants were very low, and we did not find a marked difference between the binding constant in buffer (*K*_b_=882±152 M^−1^) and that in 0.5 M TMAO (*K*_b_=592±220 M^−1^) within the experimental uncertainty, the *K*_b_ value in TMAO solution seems to be slightly smaller, however. Furthermore, the application of pressure had only a minor effect on the binding constant at both solution conditions (e. g., *K*_b_=1530±350 M^−1^ in buffer, *K*_b_=997±235 M^−1^ in 0.5 M TMAO for *p*=500 bar). These data indicate that the high pressure‐sensitivity of the γ‐globulin condensate is not reflected by the strength of the pairwise γ‐globulin interactions in the diluted phase.

**Figure 4:**
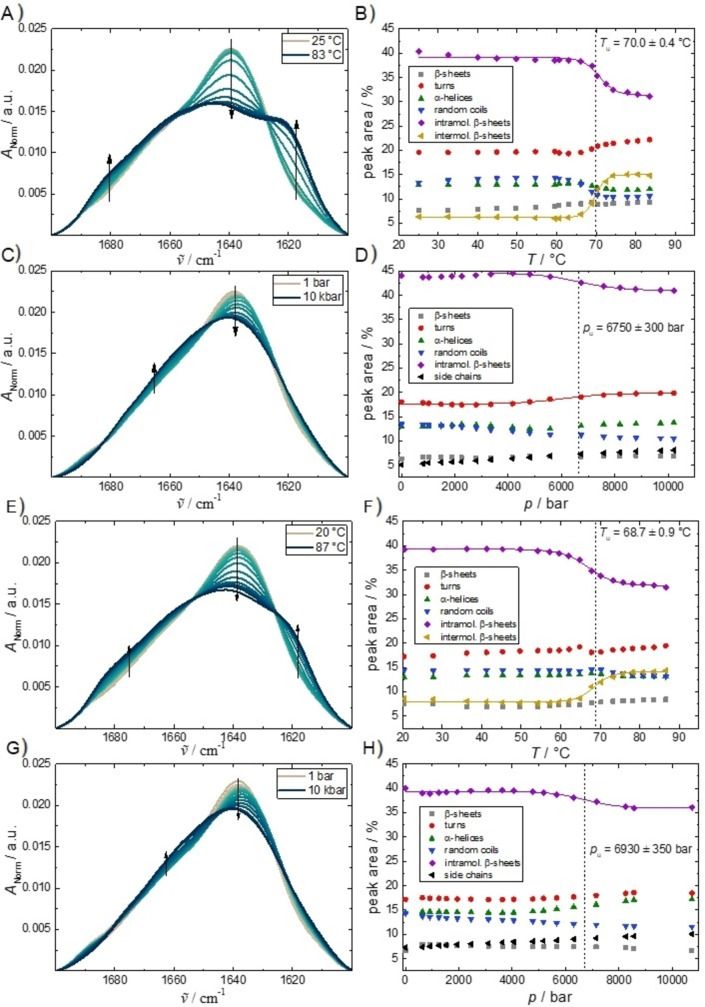
Temperature‐ and pressure‐dependent FTIR absorption data of γ‐globulin (IgG (∼80 %), IgM (∼10 %) and IgA (<10 %)) in neat buffer (A–D) and in the presence of 10 wt % PEG 1000 (E–H) leading to formation of the LLPS state at low temperatures and pressures. Left: normalized amide I′ band, right: respective secondary structure elements of temperature‐ and pressure‐induced changes. The temperature‐dependent measurements were recorded at ambient pressure (1 bar), the pressure dependent measurements at *T*=21 °C. The absolute values of secondary structure elements obtained for different sample preparations may differ by a few percent owing to the high background of the diamond anvil cell.

The next step was to explore if the conformation of the protein changes upon droplet formation and how the secondary structure of γ‐globulin is affected by temperature and pressure. To this end, the conformation‐sensitive amide‐I’ infrared band was analyzed by FTIR spectroscopy using a peak‐fitting routine that yields quantitative information about the respective fractions of secondary structure elements of the protein. The thermal stability of γ‐globulin has already been investigated by CD and DSC measurements. γ‐globulin has two domains, two Fab and Fc regions, which denature independently followed by an irreversible aggregation step at about 70 °C.[^35^, ^36^] Figure 4A, B shows the temperature dependence data of γ‐globulin at ambient pressure. Seven FTIR sub‐bands have been identified (located at ∼1683, ∼1666, ∼1654, ∼1644, ∼1634 & 1622, and 1616 cm^−1^), which correspond to β‐sheets, turns/loops, α‐helices, random coils, intramolecular β‐sheets, and intermolecular β‐sheets (of aggregates), respectively. Up to ∼65 °C, the secondary‐structure elements remained essentially unchanged, the intramolecular β‐sheet content being the dominating fraction, in agreement with literature data.^[37]^ Above 65 °C, the amount of intramolecular β‐sheets started to decrease, whereas the content of turns and intermolecular β‐sheets (being characteristic for protein aggregation) began to increase concomitantly. This means that as the temperature increases, the protein simultaneously begins to unfold and forms aggregates. Sigmoidal fits of the absorbance shifts yielded an unfolding temperature, *T*_m_, of 70.0±0.4 °C, in good agreement with the DSC and literature data.[^35^, ^36^, ^37^] Of note, due to the aggregation process following protein unfolding, temperature denaturation is partially irreversible, which prevents using *T*_m_ values (if proportional to the Gibbs free energy change) as a measure of the true thermodynamic stability of the system. Conversely, all pressure‐dependent studies are generally fully reversible.[^23^, ^24^, ^25^, ^26^]

Figure 4C, D shows the pressure dependence of the secondary structure of γ‐globulin in Tris buffer up to 10 kbar for *T*=21 °C. Up to ∼5000 bar, no pressure‐induced change could be detected. From ∼5000 to ∼7000 bar, a loss of ∼4 % in the intramolecular β‐sheet content was determined only, with a concomitant increase in turn and loop structures, indicating a partial pressure‐induced unfolding of the protein. The midpoint pressure of this partial unfolding, *p*_m_, was determined to 6750±300 bar. In contrast to the temperature‐dependent measurements, no aggregation occurs upon pressurization, and the loss in native secondary structure is much less pronounced. This partial pressure‐induced denaturation is accompanied by a volume change, Δ*V*, of −28±2 mL mol^−1^, as determined from the pressure‐dependence of the equilibrium constant, (dln*K*/d*p*)_*T*_=−Δ*V*/(*RT*),^[16]^ for this conformational transition. High pressure resistivity of the structure of human antibody immunoglobulin G up to 5 kbar was also concluded from recent small‐angle X‐ray scattering (SAXS) experiments; the radius of gyration showed a slight increase at elevated pressures, only.^[38]^ In the presence of 0.5 M TMAO (Figure S3), no significant changes of the temperature (*T*_m_=71.4±0.6 °C) or pressure (*p*_m_=6450±150 bar) stability of the γ‐globulin was observed.

Figure 4E–H also shows the FTIR spectra of the protein in the LLPS state, that is, in the presence of 10 wt % PEG 1000, where the spectra are expected to be dominated by the secondary structure of the protein molecules embedded in the droplet phase owing to their much higher concentration. As can be clearly seen, no significant secondary structural changes were observed compared to the antibody in dispersed solution. The behavior at higher pressures and temperatures in the presence of 10 wt % PEG 1000 was found to be almost identical to that of the PEG‐free diluted system. By taking the lower and upper part of the solution in the phase‐separated LLPS state at low temperatures and pressures, FTIR spectra were also recorded for the protein‐poor and protein‐rich phase separately. The data and their analyses are shown in Figure S4. Again, these data indicated that the secondary structure elements of the protein do not change significantly in the droplet phase compared to the dispersed diluted phase within the accuracy of the data.

## Conclusions

Highly concentrated antibody solutions (∼100 mg mL^−1^) are often required for formulations; this presents several challenges during preparation, including long‐term stability and shelf life. At high concentrations, the increased intermolecular interactions can lead to an increased tendency for phase separation of the solution and subsequent crystallization or aggregation and amyloid‐like fibril formation. Many excipients with different physicochemical properties have been used, such as sorbitol, sucrose, and amino acids, to improve the colloidal stability of solutions. The mechanisms by which excipients affect the stability of protein solutions can be very complex, changing attractive interactions between protein molecules directly through excipient‐protein interactions or indirectly by altering the hydration properties of the protein and the activity (coefficients) of all components, but their effects are generally difficult to predict.[^6^, ^10^] Measured excipient preferential interaction coefficients revealed interaction parameters that are generally indicative of thermodynamically unfavorable protein‐excipient interactions (negative preferential binding coefficients, *Γ*_PC_, between protein and cosolvent), although specific interactions with the protein interface might still occur.[^6^, ^27^, ^28^] Here, we have seen that the compatible cosolvent TMAO had no drastic effect on the temperature and pressure stability of the droplet phase even at the high concentration of 0.5 M, leading only to a slight destabilization of the droplet phase. A different scenario was observed in other LLPS systems, such as γ‐crystallin and SynGAP/PSD‐95, where a strong stabilization of the droplet phase was observed, which was assumed to be largely devoid of the cosolvent.[^15^, ^19^, ^20^] The slight destabilization of the droplet phase of γ‐globulin by TMAO is probably due to some specific protein‐excipient interactions, as suggested by the decrease of the *K*_b_ value in the TMAO solution.

In this work, we have explored a different approach to modulate LLPS formation and to suppress subsequent aggregation and precipitation of immunoglobulins. We have shown that the liquid‐phase droplets of γ‐globulin are very pressure‐sensitive biomolecular assemblies. Increasing the pressure by several tens of to hundred bar leads to a drastic decrease in the droplet stability in buffer solution, with complete disappearance of the droplet phase beyond about 400 bar. Lower pressures are required in TMAO solution. The pressure dependence of the pairwise γ‐globulin association is not enough to explain the observed pressure sensitivity of the droplet phase. So what is the reason behind the pronounced pressure sensitivity of the LLPS phase of the globulin solution?

**Figure 5:**
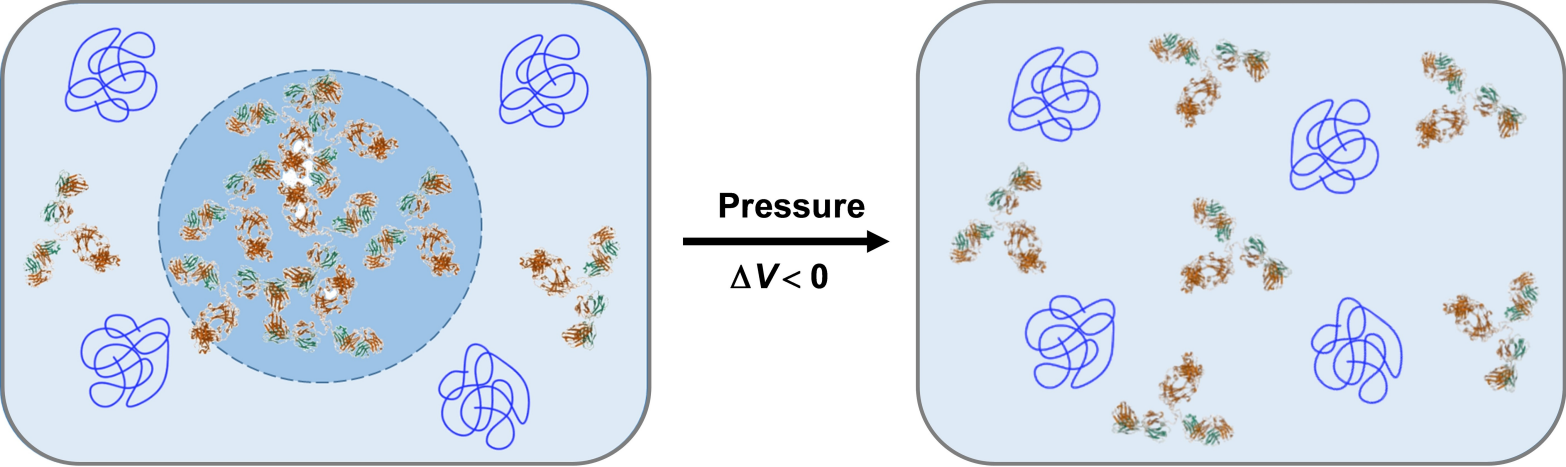
Schematic depicting the proposed mechanism of pressure‐induced dissolution of γ‐globulin droplets (PDB ID 1IGT was used for the structure of IgG). The light and heavy chains of the immunoglobulins are colored green and brown, respectively. The crowding agent PEG 1000 is represented as blue coils and (transient) void volumes in the droplet phase are shown in white.

The LLPS formation of antibodies implies strong attractive enthalpic protein‐protein interactions through multiple charge–patch interactions of complementary surface charge, which require greater than room temperature (here, *T* >30 °C) for the entropic contribution to the Gibbs free energy of mixing to dominate and favor a homogeneous solution.[^4^, ^5^, ^10^] Abs have a more branched and flexible structure than many other proteins, such as the globular lysozyme molecule. As a result, the binodal of LLPS of antibodies has a much lower critical volume fraction than globular proteins because of their nonspherical and anisotropic nature. The observed low critical volume fraction, *ϕ*_crit_, is likely the result of a large effective exclusion volume of Ab molecules (*ϕ*_crit_(Abs)≈7 %, *ϕ*_crit_(lysozyme)≈17 %). Using quasi‐elastic neutron scattering, Girelli et al. observed a strong decrease in antibody diffusion in the droplet state, although internal flexibility, which is dominated by the diffusion of the lobes at low temperatures, persists to a significant degree.^[9]^ Hence, being large, branched and flexible protein molecules, Abs can be assumed to generate a significant amount of anhydrous void volume when packed in the dense droplet phase. Therefore, a plausible explanation is that there is a considerable (transient) void volume inaccessible to water molecules linked to the multiple‐protein molecule interaction network of the condensate compared to the dispersed phase of γ‐globulin. According to Le Châtelier's principle, this leads to an overall decrease in the volume of the system upon dissociation of the droplet phase by pressure (Figure 5), which is also favored by a higher mixing entropy. Differential changes in protein surface hydration could make an additional contribution,^[40]^ which is probably of minor importance here. Of note, void volumes can also arise from imperfect packing in folded structures of globular proteins, but these unfold at much higher pressures, typically between ∼4 and ∼8 kbar.^[39]^

In this study, we assessed the practicality of employing high pressure as a technique to suppress LLPS formation and the aggregation of concentrated antibody solutions. Some other practical applications of the high‐pressure technology have already proven successful. Pressure treatment at kbar pressures (4–6 kbar) has a long history in high‐pressure food processing, which is an example of a well‐established biotechnological application. High hydrostatic pressure has the potential to inactivate microorganisms, viruses, and enzymes, while having only a small effect on the flavor and nutritional content of food compared to the effects of thermal treatments.[^25^, ^26^, ^41^, ^42^] High‐pressure‐treated products include juices and milk. Milk is naturally high in antibody content, which is desirably preserved during processing. In fact, immunoglobulins form an important component of the immunological activity found in milk and colostrum.^[43]^ They are also central to the immunological link that occurs when the mother transfers passive immunity to the offspring. Also in bovine colostrum, the antibody system confers passive immunity until the calf‘s own immune system matures. The first colostrum contains very high concentrations of immunoglobulins (40–200 mg mL^−1^), which decrease after a few days. Here, we have shown that the immunoglobulin molecules themselves are very resistant to unfolding under high pressure, even up to about 6 kbar, that is, they are expected to be resistant against the pressure treatment of milk.

## Experimental Section

**Sample preparation**: Bovine γ‐globulin (G5009), containing ∼80 % IgG, ∼10 % IgM, and <10 % IgA, was purchased from Sigma‐Aldrich and dissolved in buffer containing 20 mM Tris (pH 7.5), 150 mM NaCl and 2 mM sodium azide. Subsequently, the protein sample was centrifuged for 10 min at 1000 rpm. To induce phase separation, the protein solution was mixed in a 1 : 1 ratio with a 20 % (*w*/*v*) PEG 1000 solution. The sample was equilibrated for 30–60 min at room temperature. For concentration determination by UV/Vis absorption spectroscopy, an extinction coefficient at 280 nm of 1.4 mL mg^−1^ cm^−1^ was used.^[44]^

**Turbidity measurements**: The UV/Vis spectrometer UV‐1800 Shimadzu was used to perform the temperature‐dependent experiments in a 3 mm quartz cuvette. To control the temperature inside the cuvette, an external water bath was utilized. The pressure‐dependent turbidity measurements at 400 nm were performed on the UV/Vis spectrometer Lambda 25 (Perkin Elmer). To this end, a home‐built high‐pressure cell with sapphire windows (diameter 20 mm, thickness 10 mm) was used, and the liquid sample was separated from liquid water as the pressurizing medium by a polymer film. The pressure inside the cell was regulated hydrostatically using a high‐pressure hand pump. To control the temperature inside the cell, an external water bath was used.

**Microscopy**: Bright‐field light microscopy images were recorded on an Eclipse TE2000‐U microscope (Nikon Inc.) using a Nikon Plan Fluor 20x objective (NA 0.45, WD 7.4). For pressure application, a home‐built pressure cell equipped with flat diamond windows was used. Pressure was applied hydrostatically with a high‐pressure hand pump.

**DSC measurements**: The DSC measurements were performed using a TA Instrument (New Castle, DE) Q20 differential scanning calorimeter. A total sample volume of 20 μL with a protein concentration of 100 mg mL^−1^ was used. As a reference, the respective buffer solution was used. Both the reference and the sample cell were heated from 1 to 90 °C at a heating rate of 1 °C min^−1^.

**Fluorescence‐labeling of γ‐globulin**: 10 mg γ‐globulin powder was dissolved in buffer containing 100 mM NaHCO_3_ (pH 8.5), 1 mM CaCl_2_, and 2 mg dansyl chloride were dissolved in 200 μL acetone. A fivefold excess of dansyl chloride was added to the protein solution. The solution was incubated rotating for 2 h in the dark. A 5 mL HiTrap Desalting column with Sephadex G‐25 resin was used to remove excess dye.

**Fluorescence anisotropy**: Fluorescence anisotropy measurements were carried out using a K2 fluorometer from ISS. As excitation wavelength, 340 nm was used, and the emission was collected at 500 nm. The excitation monochromator was set at 8 nm and the emission monochromators at 4 nm. For the pressure‐dependent measurements, an ISS high‐pressure cell with 10 mm thick quartz windows was used. By means of an external water bath, the temperature inside the pressure cell was kept constant at 20 °C during the measurements. A small quartz cuvette filled with the sample solution was sealed with DuraSeal^TM^ laboratory stretch film and placed inside the high‐pressure cell. Pressure was applied hydrostatically using a manual pump and water as pressurizing medium. The concentration of the fluorescently labeled γ‐globulin was 0.43 μM for each measurement, whereas the concentration of unlabeled γ‐globulin was varied in a range from 0 to 250 μM. Application of high pressure can cause depolarization of light due to a scrambling effect on the high‐pressure cell‘s quartz windows. However, at the pressures employed during these measurements, the scrambling effects of the optical windows are negligible. Thus, no anisotropy corrections were needed.^[45]^

**FTIR‐spectroscopy**: γ‐globulin from bovine blood (Sigma‐Aldrich, SRE0011) and PEG 1000 (Carl Roth) were dialyzed against D_2_O using Amicon Ultra (2 mL) centrifugation units with 10 kDa cutoff, and subsequently lyophilized and purified by dialysis to remove the additives. Trimethylamine‐*N*‐oxide (TMAO), Tris base (tris(hydroxymethyl)aminomethane), NaCl and NaN_3_ were obtained from Sigma Aldrich and used without further purification. The antibody/PEG solutions were dissolved in Tris (D_2_O) buffer (20 mM Tris, 2 mM NaN_3_ and 150 mM NaCl). The pD for each solution was adjusted to 7.0 by adding DCl. A protein concentration of 8 wt % and 10 wt % PEG was used in Tris (D_2_O) buffer solution. The droplet phase was prepared in the same way as for the microscopy measurements. Both temperature‐ and pressure‐dependent FTIR measurements were performed using a Nicolet 6700 (Thermo Fisher Scientific) spectrometer equipped with a liquid‐nitrogen cooled MCT‐detector (HgCdTe) and data recorded in the wavenumber range between 4000 to 650 cm^−1^. The required temperature in the cell was controlled to 0.1 °C using an external circulating water thermostat. High pressures (1 bar–10 kbar) could be achieved with a membrane‐driven diamond anvil cell (Diacells VivoDac, Almax easyLab), equipped with type IIa diamonds (Almax easyLab), which was connected to an automated pneumatic pressure controller (Diacells iGM Controller, Almax easyLab). The pressure equilibration was maintained for 5 min and temperature equilibration for 10 min before collecting the spectra. BaSO_4_ powder was used as an internal pressure calibrant to determine the pressure values. BaSO_4_ shows a characteristic pressure sensitive symmetric stretching mode around 983.5 cm^−1^ which increases linearly with pressure.^46^ The sample chamber of the FTIR spectrometer was continuously purged with CO_2_‐free dry air to achieve a good signal‐to‐noise ratio. 128 scans in a row were recorded for each spectrum. The spectra were processed with Happ–Genzel apodization using the Omnic 7.2 spectral processing software. The spectral analysis was carried out using the Thermo Grams 8.0 software. After buffer subtraction and smoothing of each spectrum, the area of the amide I' band (1700–1600 cm^−1^) was normalized to 1. The numbers and positions of the sub‐bands were determined by using two mathematical operations, Fourier self‐deconvoluted (FSD) and 2nd derivative spectroscopy, to help identify the position of secondary structure elements and evaluate conformational changes. Here, the amide I’ band region of the antibodies could be divided into seven sub‐bands.^[37]^ The relative changes in the population of secondary structure elements were obtained by using mixed Gaussian–Lorentzian line‐shape functions in the fitting procedure.^[47]^

## Conflict of interest

The authors declare no conflict of interest.

1

## Supporting information

As a service to our authors and readers, this journal provides supporting information supplied by the authors. Such materials are peer reviewed and may be re‐organized for online delivery, but are not copy‐edited or typeset. Technical support issues arising from supporting information (other than missing files) should be addressed to the authors.

Supporting Information

## Data Availability

The data that support the findings of this study are available from the corresponding author upon reasonable request.
