# Supplementary material for: Suppression of Liquid‐Liquid Phase Separation and Aggregation of Antibodies by Modest Pressure Application
Source: Chemistry. 2022 Jul 18;28(48):e202201658. doi: 10.1002/chem.202201658 (PMC9544093; doi:10.1002/chem.202201658)
Supplement: Supplementary file 1 — Supporting Information [file CHEM-28-0-s001.pdf]

Chemistry–A European Journal

Supporting Information

Suppression of Liquid-Liquid Phase Separation and Aggregation of Antibodies by Modest Pressure Application

Zamira Fetahaj, Michel W. Jaworek, Rosario Oliva, and Roland Winter*

Additional Figures

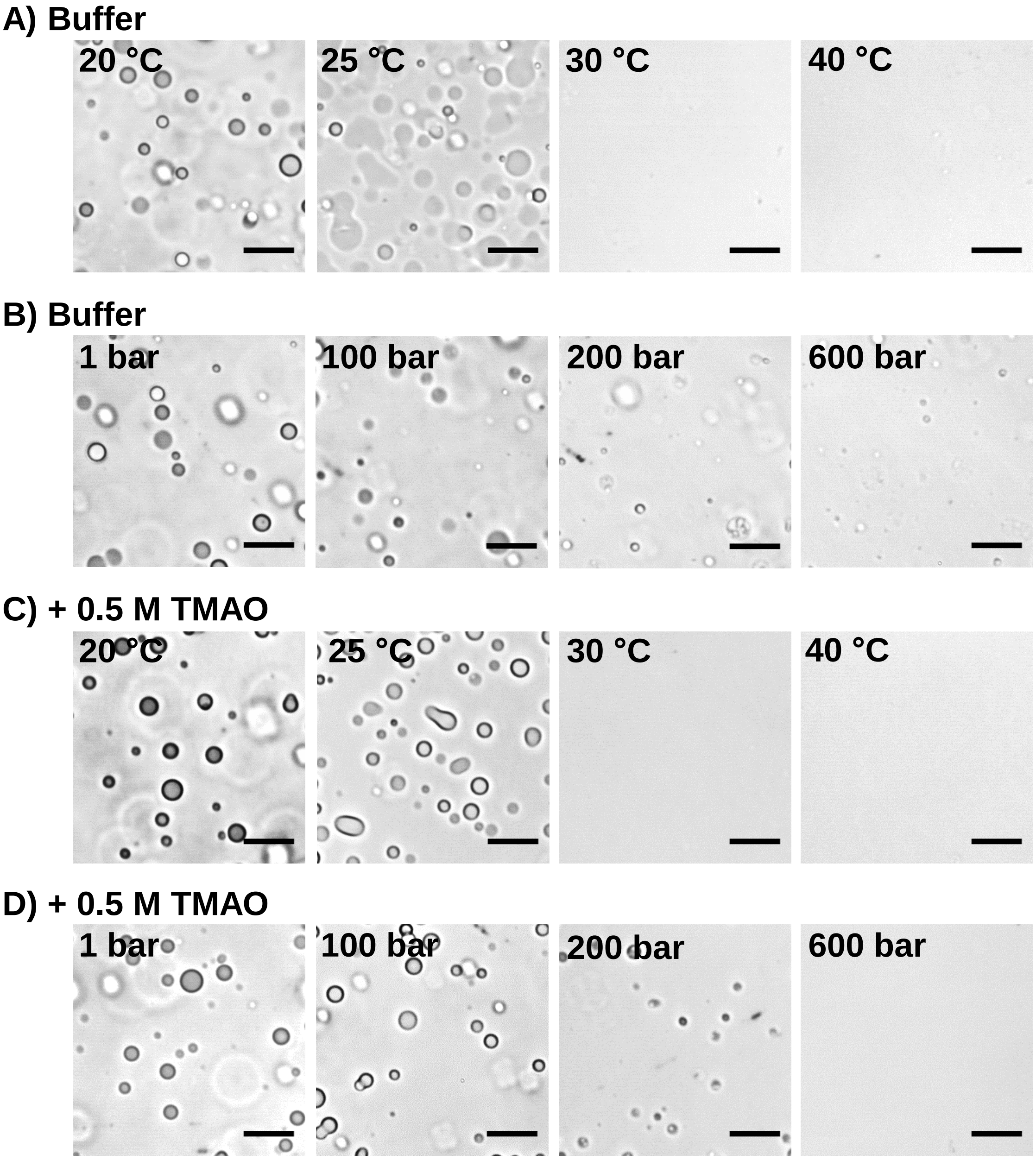

Figure S1. Microscopy snapshots of γ-globulin's LLPS on the bottom window of the used microscopy cell. (A) Temperature and (B) pressure dependence (at T = 20 °C) in buffer. (C) Temperature and (D) pressure dependence (at T = 20 °C) in the presence of 0.5 M TMAO. Scale bar = 20 µm.

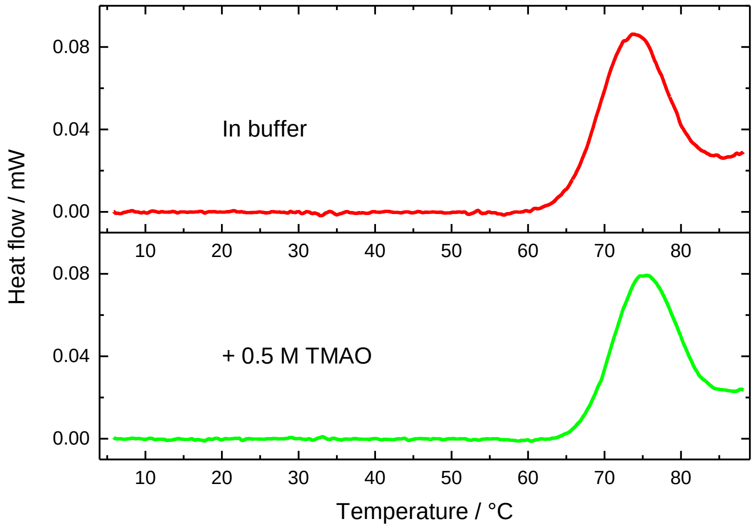

Figure S2. DSC thermograms of γ-globulin in buffer and in the presence of 0.5 M TMAO. 2 mg of protein were heated from 1 to 90 °C at a heating rate of 1 °C min^{-1}.

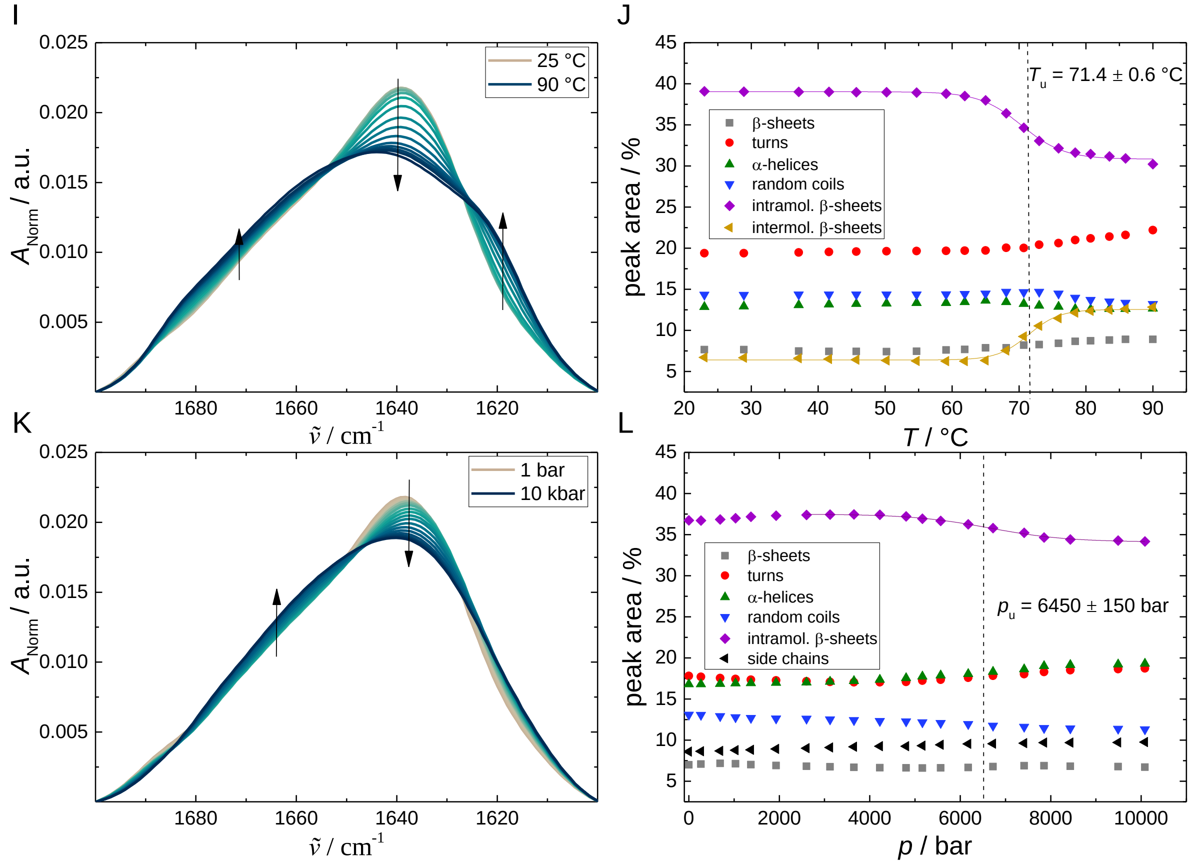

Figure S3. Temperature- and pressure-dependent FTIR absorption data of 8 wt% γ-globulin (IgG (~ 80%), IgM (~10%) and IgA (<10%)) in the presence of 0.5 M TMAO (I,K), The temperature dependent measurements have been carried out at ambient pressure, the pressure dependent measurements at *T* = 21 °C, I: temperature-induced changes of the normalized amide I´ band, G,K: pressure-induced changes of the normalized amide I´ band, and the corresponding changes of secondary structure elements are shown on the right-hand side.

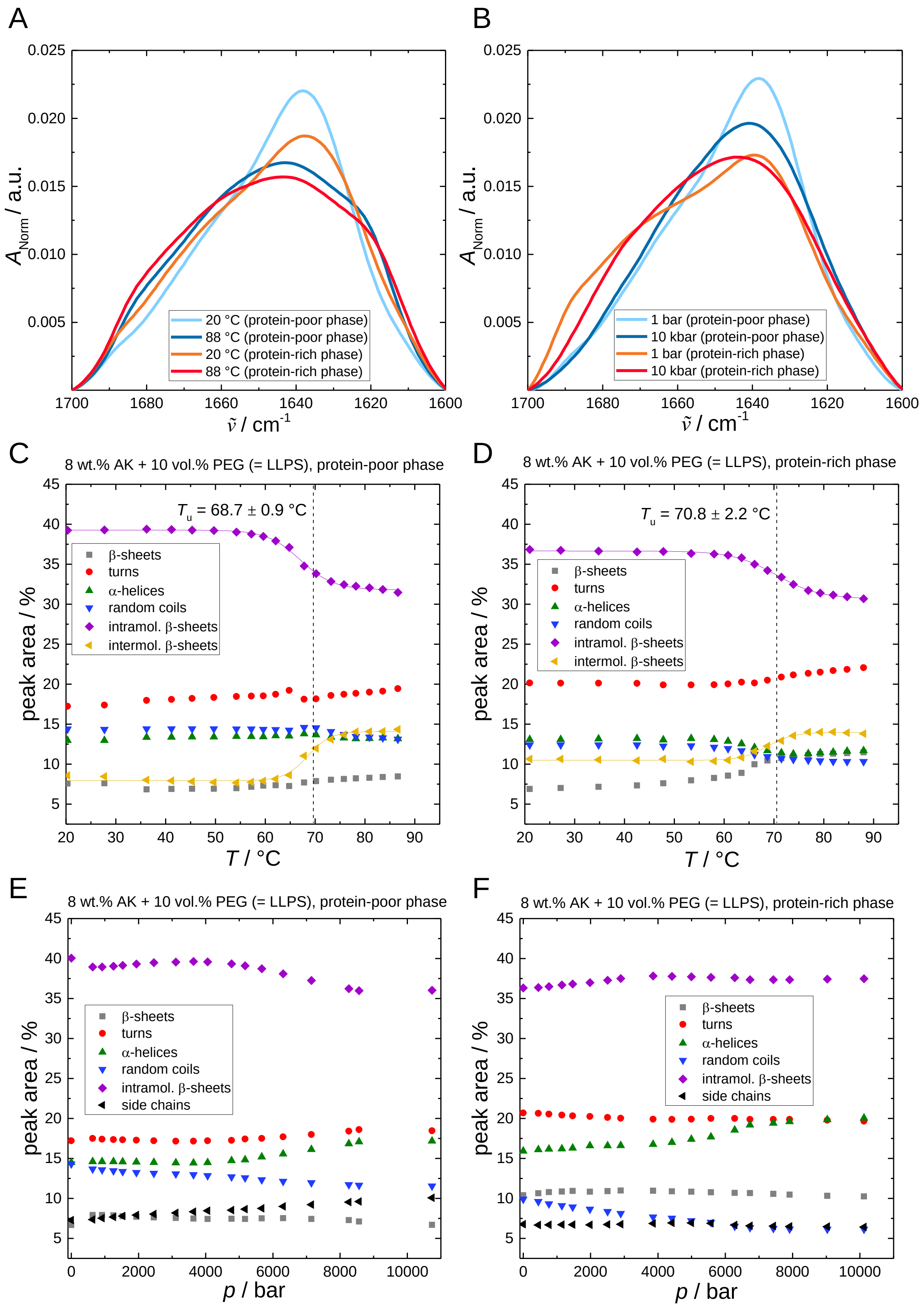

Figure S4. Temperature- and pressure-dependent FTIR absorption data of γ-globulin in the protein-poor and protein-rich phase. Temperature-induced (A) and pressure-induced changes (B) of the normalized amide I´ band and the corresponding changes of secondary structure elements (C-F).
